# School Attendance Following Receipt of Care From a School-Based Health Center

**DOI:** 10.1016/j.jadohealth.2023.07.012

**Published:** 2023-09-13

**Authors:** Caryssa Lim, Paul J. Chung, Christopher Biely, Nicholas J. Jackson, Maryjane Puffer, Alex Zepeda, Patricia Anton, Kathryn M. Leifheit, Rebecca Dudovitz

**Affiliations:** a Department of Health Systems Science, Kaiser Permanente School of Medicine, Pasadena, California; b Departments of Pediatrics and Health Policy & Management, University of California Los Angeles, Los Angeles, California; c Department of Medicine, University of California Los Angeles, Los Angeles, California; d The L.A. Trust for Children’s Health, Los Angeles, California; e Anton Consulting, Los Angeles, California

**Keywords:** Schools, School-based health centers, Mental health

## Abstract

**Purpose:**

School-based health centers (SBHCs) may mitigate barriers to health care access and improve students’ academic outcomes, but few studies test this hypothesis. We examined whether school attendance improved after students received care at an SBHC.

**Methods:**

We conducted a secondary analysis of data from 17 SBHCs affiliated with a single large urban school district and demographic and attendance data from SBHC users (N = 14,030) and nonusers (N = 230,046) from August 2015–February 2020. We examined the percent of full school days present each month for three years before and after students’ first SBHC visit and a proxy visit date for SBHC nonusers. Propensity weighted linear regression models tested whether visiting an SBHC was associated with a change in the attendance trajectory compared to a matched sample of SBHC nonusers.

**Results:**

Among SBHC users, attendance trajectories declined more steeply prior to their first SBHC visit than after the first visit (preslope −0.71%, postslope −0.05%), whereas SBHC nonusers had a similar attendance trajectory over the entire period (preslope −0.18%, postslope −0.17%), with difference-in-difference 0.65. Changes in trajectories were more pronounced for students with a mental health encounter. Prior to the first SBHC mental health visit, SBHC users displayed a marked decline in monthly attendance (preslope −1.02%). After the first mental health visit, attendance increased (postslope 1.44%), with difference-in-difference 2.33.

**Discussion:**

SBHC utilization was associated with improved school attendance over time, particularly for students with a mental health diagnosis. Investing in SBHCs may reduce school absenteeism and support student health.

School-based health centers (SBHCs) provide primary care, mental health care, and other essential health services to students, families, and community members. From 1998 to 2017, the number of centers in the United States doubled from 1,135 to 2,584, and the number continues to rise [1]. Studies show that SBHCs increase access to care, particularly for underserved groups, including individuals without health insurance and those of low socioeconomic status [2]. By placing health services conveniently within schools, SBHCs remove significant barriers to health care access for children and their families, including transportation and cost. In addition, SBHCs are thought to support children’s school function by addressing health concerns that might get in the way of students’ academic success without requiring them to leave campus and miss school [3,4].

SBHCs may be particularly important in the context of the United States’ youth mental health crisis. Rates of psychological distress have soared during the Covid-19 pandemic, with a recent meta-analysis estimating that pediatric depression and anxiety doubled during this time [5]. Poor mental health, in particular, is consistently associated with poor short- and long-term academic and health outcomes [3,6]. With almost half of children not receiving the mental health care that they need, many have called upon school-based health centers to be an appropriate setting for providing mental health services [7]. In 2015, the National Survey on Drug Use and Health reported that 3.2 million adolescents in the United States received mental health services in an educational setting [8]. Students with mental health need have lower school attendance, with mental health-related reasons as the major reason for absences from school [9]. In recent years, we have seen a dramatic worsening of chronic absenteeism [10] and a renewed interest in investing in SBHCs’ capacity to address mental health needs, in the hopes that this leads to both improved adolescent wellbeing and improved school attendance.

In several smaller-scale, descriptive studies, SBHC utilization appears correlated with improvements in academic outcomes, including improved grades, graduation and promotion rates, attendance, and reduced suspensions, primarily for high-risk adolescents, such as pregnant and parenting students [2,11]. In two separate studies in New York, authors found that students enrolled in SBHCs had better attendance, more time spent in class on average, and reduced rates of asthma-related hospitalizations compared to non-SBHC users [12,13]. Meanwhile, a study comparing school attendance between ninth grade SBHC-users and non-users in Seattle showed mixed results [14]. School attendance is critical for student achievement and educational attainment, both powerful social determinants of health, and may also have implications for school funding, such as in states where funding is tied to daily attendance counts [15]. SBHCs are hypothesized to support school attendance by addressing physical and mental health needs driving school absenteeism, providing care without necessitating a school absence, and increasing school connectedness [12].

However, there are limitations to previous studies. First, while SBHC utilization has been shown to be associated with improved school attendance, associations between SBHC utilization and attendance are likely heterogeneous depending on types of services received. In particular, visits specifically for mental health concerns may be more strongly associated with improved outcomes than other types of visits. Studies to-date have been unable to explore heterogeneity by SBHC service type. Additionally, a systematic review of SBHC impact on academic outcomes found that the majority of the analyses were conducted at the school and district-level, not at the individual-level [16]. While the presence of SBHCs on school campuses may infer some insight to student use, it cannot act as a true proxy for utilization to services. These limitations are largely due to privacy and confidentiality issues related to connecting educational data with SBHC health center data [2]. Finally, many studies are limited by sample size and lack of a matched control group [11]. This study expands upon existing literature by utilizing a large, robust dataset that links health and education data at an individual student level to test whether SBHC utilization is associated with improved attendance. We recognize, however, that although school attendance would be expected to increase due to reductions in illness, it is also possible that parents and caregivers may send their children to school to receive medical care at SBHCs and therefore increase attendance due to illness—not due to improved health [2].

Understanding how student use of school-based health centers impacts academic performance is critical for estimating potential academic and health returns on investment for SBHC across the United States. In this study, we examined whether school attendance improved after students received care at an SBHC.

## Methods

### Data source

We conducted a secondary analysis of de-identified data from The Los Angeles Trust for Children’s Health’s Data xChange. This dataset contains encounter data extracted from electronic health records from 17 school-based health centers affiliated with a large urban school district in Southern California and demographic and attendance data from the participating school district on all enrolled students from July 1, 2015-March 31, 2021. Academic and health outcomes are linked at the individual student level and then de-identified for analysis. There has been one previous study published using this data set, which described SBHC utilization during COVID-19 pandemic-related school closures [17]. For this analysis, we included data from August 2015 to February 2020, as both school attendance and health care utilization changed substantially due to COVID-related school closures at that time [18]. The UCLA Institutional Review Board (IRB) reviewed the study and determined that it was exempt.

### Measures

Our primary outcome was attendance rate, defined as the percent of full school-days present each month during the school year and calculated using the number of full-days present as the numerator and the sum of full days present, full days absent, and partial days absent each month for the denominator. We excluded the summer months because access to summer school was not universal during the observation period and the drivers of attendance during summer school may differ from those during the traditional school year. We then multiplied this by nine to approximate the annual attendance over a 9-month school year.

Time was modeled as a continuous variable relative to date of a student’s first visit to an SBHC. The date of the first SBHC visit constituted time = 0 with all dates prior to this point having a negative value and all dates after this point having a positive value. We also constructed time relative to the first SBHC encounter associated with a mental health diagnosis, per the ICD codes associated with each encounter. Some common diagnoses for which students sought care included depression, anxiety, autism, and attention-deficit/hyperactivity disorder. Although some of the participating SBHCs offer mental health care through the participating school district or a third-party provider, this approach can only account for mental health visits with the SBHC provider.

Demographic variables were obtained from and defined by the school district and included only sex, and race/ethnicity. Sex was defined as a categorical variable with options for male and female, as recorded on students’ official school record. Race/ethnicity was defined as a categorical variable, with options for Asian, Black, Latinx, White, or two or more.

### Proxy date assignment

For those who did not visit an SBHC, we applied a proxy date for SBHC attendance [19]. This proxy date was based on matching the distribution of sex and date (month and year) when first appearing in the attendance dataset to those who visited the SBHC. Date first appearing in the dataset was selected, as it correlates with both age and length of time in the dataset. Within these matched subsets, we randomly applied a proxy SBHC first visit date that had the same distribution as the first dates of the SBHC visitors. In this manner, we ensured that the sex distribution, length of time in the dataset, and distribution of SBHC first attendance dates (or proxy) were exactly the same in the exposed and unexposed groups. See our methods supplement for more details on this procedure. A similar process was used to construct proxy dates for a first SBHC mental health visit.

### Propensity score development

In addition to matching the sex distribution and between those who did and did not visit an SBHC, we developed a propensity score weight based on race, sex, and their interaction in predicting their use of an SBHC. This propensity score was converted to an inverse probability weight and used in all subsequent analyses. A similar process was used to construct a propensity score for an SBHC mental health visit.

### Statistical analysis

To examine if the rate of change in attendance differed between SBHC users and nonusers, we utilized a linear regression model with clustered robust standard errors for student to account for within-student correlation over time. Main effects for time period (before vs. after visiting an SBHC or proxy date), months since visiting an SBHC (modeled linearly), and exposure group (SBHC user versus. nonuser) along with all possible two-way and three-way interactions between these variables were specified. The change in slope for attendance before versus after a first visit was compared between SBHC and non-SBHC groups using the three-way interaction term in a difference-in-difference estimate of trend lines. We limited the timeframe in our main analysis to within 36 months of visiting an SBHC or proxy date. A 36-month timeframe was used here due to sparse data at more distant time points, as well as a priori hypothesis that SBHC utilization would mainly affect attendance in the short- and intermediate-term. Sensitivity analyses were constructed to test the robustness of results to this restriction by A) including all attendance observations, including those outside of this 36-month window and B) excluding those with missing data during the six school-month periods before and after visiting the SBHC or proxy date. This second sensitivity analysis was conducted to ensure our findings were not driven by differences in the student population over time, as high-risk students dropped out or left the school district, resulting in a student population that would be more likely to have good school attendance over time. All analyses were conducted using the previously described inverse probability weight.

Similar models tested whether a first SBHC encounter for mental health services was associated with a change in attendance.

## Results

There were 14,030 students who visited an SBHC at least one time. Within this sample of SBHC users, 983 students visited specifically for a mental health concern (Table 1). Among students who visited an SBHC for any reason, nearly 77% self-identified as Latinx, followed by White (10.5%), Black (6.0%), Asian (5.9%), and two or more (0.2%). There were more female students accessing SBHCs (56.5%) than male students (43.5%). As described above, SBHC nonusers were matched with users on these characteristics.

**Figure 1.**
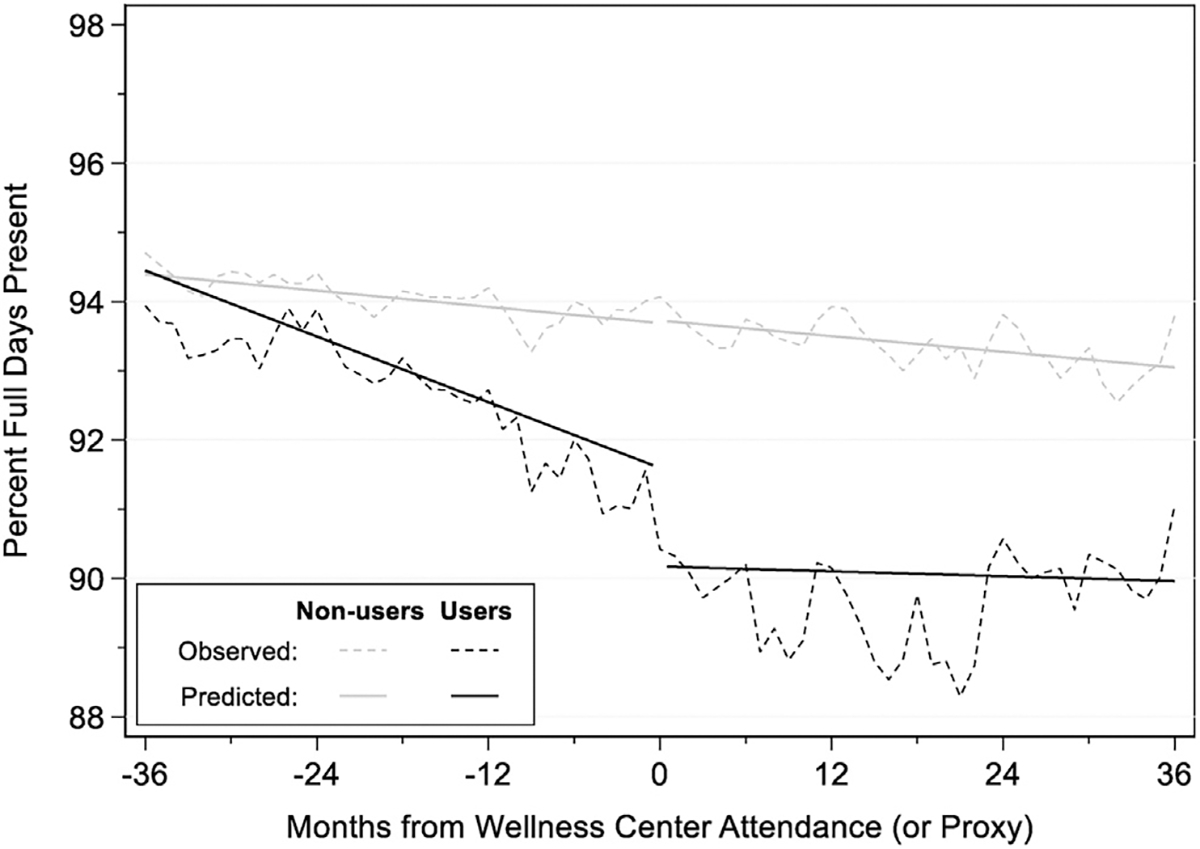
Change in Attendance Following the First SBHC Visit or Proxy Date for SBHC Users and Non-Users.

As seen in Table 2 and Figure 1, on average SBHC users had a negative attendance trajectory prior to a first SBHC visit (slope = −0.71% per nine months, 95% CI −0.85, −0.58). This trajectory then stabilized after the first visit (slope = −0.05, 95% CI −0.25, 0.14). The difference between these slopes was 0.66 (95% CI 0.42, 0.91). This corresponds to an average of 0.015 additional school days per month for each SBHC user, amounting to 210 additional school days per month across the entire population of SBHC users. In comparison, SBHC nonusers had a similar attendance trajectory over the entire period (preslope = −0.18, 95% CI −0.20, −0.15; postslope = −0.17, 95% CI −0.20, −0.14; difference = 0.01, 95% CI −0.03, 0.04). The difference-in-differences between SBHC users and nonusers was significant (*p* < .001), indicating that visiting an SBHC was associated with a 0.65 percentage-point increase in school days attended per month.

**Figure 2.**
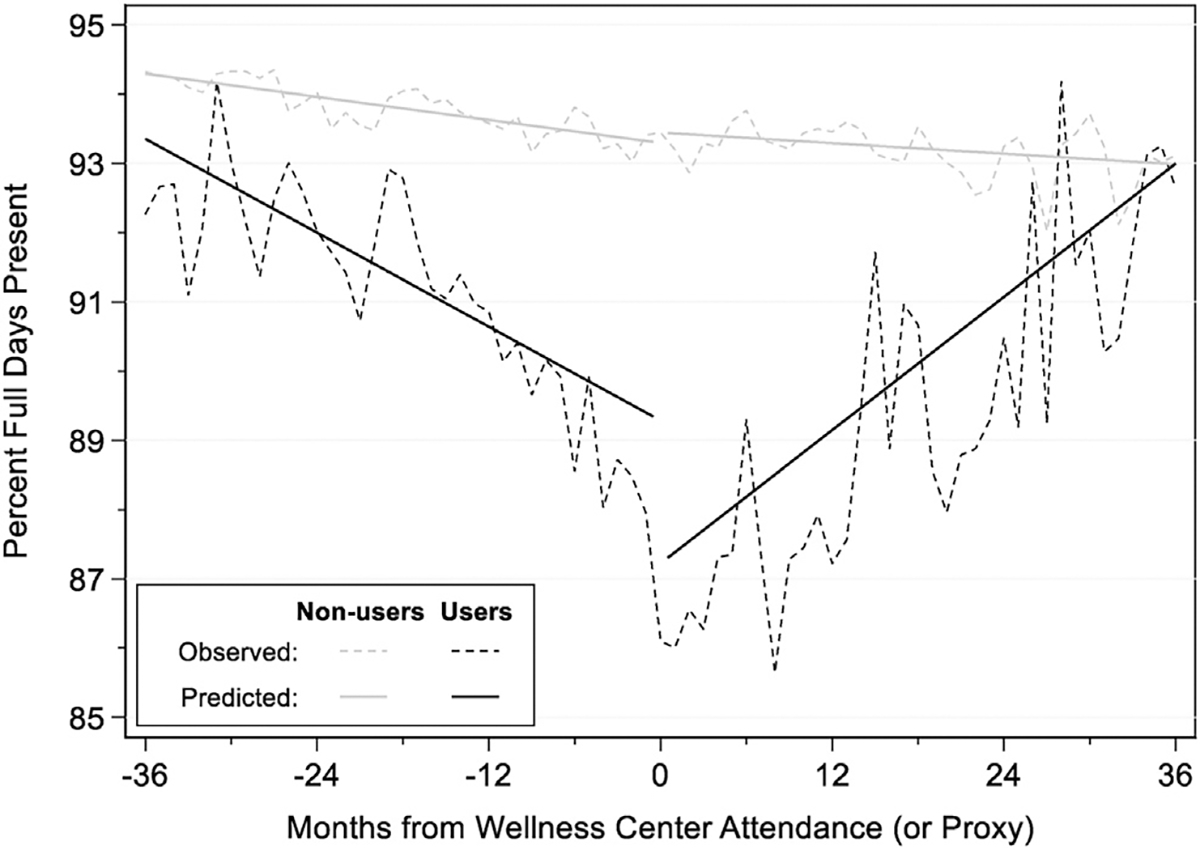
Change in Attendance Following the First SBHC Mental Health Visit or Proxy Date for SBHC Users and NonUsers.

Differences in attendance trajectory were larger for students with a mental health encounter (Table 2, Figure 2). Prior to the first SBHC mental health visit, SBHC users displayed a marked decline in monthly attendance (slope = −1.02, 95% CI −1.58, −0.46). After the first SBHC mental health visit, attendance improved with a positive slope (slope 1.44, 95% CI 0.80, 2.09). The difference in slopes was 2.46 percent of school days per 9-month school year (95% CI 1.52, 3.40). This corresponds to 0.055 additional school days per month per student, which is 5 times greater than for students who visited an SBHC for any reason. SBHC mental health nonusers demonstrated a negative slope (i.e. declining school attendance) before (slope = −0.25, 95% CI −0.28, −0.22) and after (slope = −0.11, 95% CI −0.16, −0.07) their proxy visit date. This difference in slope was 0.13 percent of school days per month (95% CI 0.08, 0.19). Hence, the difference-in-differences indicates that an SBHC mental health visit was associated with a 2.33 percentage point increase in school days attended per month (*p* < .001).

These results were similar and remained statistically significant in all sensitivity analyses (Tables A1 and A2).

## Discussion

The purpose of this study was to examine school attendance after receiving care at an SBHC. The results indicate that visiting an SBHC is significantly associated with improved attendance over time. More dramatic improvement was observed for students with a mental health diagnosis, which is consistent with previous studies [20]. These findings rigorously confirm that SBHCs may serve an important role in improving both access to care and school attendance. Given the importance of attendance for both school funding and educational attainment, a critical social determinant of health, findings suggest SBHCs may be one way to improve population health and health equity.

It remains unclear the exact mechanism by which SBHCs can improve attendance rates in students. However, a few hypotheses emerge based on existing literature. One possibility is that by reducing barriers to care, SBHCs can address underlying health conditions that lead to absenteeism [21]. Another hypothesis is that SBHCs address health conditions that inhibit students’ ability to succeed academically (e.g., mental and behavioral health conditions, distractions due to chronic conditions). Improved school function may then motivate improved attendance. Finally, previous articles have hypothesized that SBHCs improve school connectedness, defined as “the belief by students that adults in the school care about their learning as well as about them as individuals,” which then leads to better school attendance [22].

We found that SBHC users exhibited slightly worse attendance trajectories prior to their first SBHC visit than nonusers, which may be consistent with prior work suggesting SBHCs tend to serve a high-risk population [23]. In addition, we observed more dramatic improvement in attendance rates for students with a mental health diagnosis. This includes both a more steeply declining attendance trajectory prior to visiting the SBHC and a more positive attendance trajectory following a first SBHC mental health visit. These findings suggest that students with a mental health need may represent a group for whom SBHCs are particularly effective for improving academic outcomes. Previous research has also demonstrated that SBHC utilization for students with mental health needs resulted in improvements in grade point averages [14].

The close relationship between student mental health and academic outcomes is an important justification for the expansion of mental health services within school settings. The United States recently passed the Bipartisan Safer Communities Act, which has committed to $1 billion in funding over five years to double the number of school-based mental health professionals and expand mental health services in schools [24]. While there are many models for school-based mental health delivery, the SBHC model studied in this analysis is one in which mental health is integrated with primary care, which has been thought to reduce stigma associated with seeking mental health care [25]. The current findings highlight the potential value of ongoing, sustained investment in school-based health centers that offer integrated mental health services, particularly given that lack of consistent funding is a longstanding barrier to achieving sustainability in mental health programming [26]. Another potential opportunity to strengthen school-based health is through the expansion of community schools, which are public schools that aim to provide neighborhood-specific services and resources for students [27]. Community schools attempt to identify needs of students that can be readily addressed in the school setting—the place where children primarily build relationships, acquire knowledge, and learn important skills. In this model, schools serve as anchor institutions, helping to connect students and their families to services that support school function. Our findings suggest SBHCs may be one such service.

The major strength of this study is the large dataset, which includes longitudinal educational and health data at the individual level and the use of a matched control group. However, the current study has some limitations. First, though we did do a separate analysis for students with a mental health diagnosis, we did not stratify the study sample by other types of services offered at the SBHCs. Future work may explore stratification by service type and examine variations by student characteristics to identify which SBHC services might be most impactful for which students. Secondly, this study specifically measured attendance following the first visit to the SBHC. We did not account for differential outcomes based on multiple visits to SBHCs. It is possible that there is a positive relationship between number of SBHC visits and magnitude of attendance improvement. Additionally, study data are limited to a single large urban school district FHQC-sponsored SBHCs. While this is the most common and fastest growing SBHC model, findings may not generalize to other contexts [1]. Our study design limits the ability to make causal inferences. First, we are unable to account for unmeasured confounding: it is possible, for example, that SBHC users received academic interventions around the same time that they visited SBHCs or that an acute, stress-inciting event could trigger both a dip in attendance and an SBHC visit. Although we used both matching and propensity weighting to account for selection into an SBHC, we were limited to demographic variables for this process, which is unlikely to fully address selection bias. Given that SBHCs are likely to serve a riskier population for whom it may be more difficult to improve school attendance, we believe this is most likely to bias our results toward the null hypothesis. However, we cannot rule out the possibility that a riskier population has more room for improvement and results reflect regression to the mean. We feel the consistent and robust pattern in attendance trajectories in the SBHC user groups and difference in this pattern relative to the control groups makes this unlikely. We also recognize there are other models for mental health care delivery in schools. The SBHC model integrates mental health within other health services, while other models use specific school-based mental health programs that are not delivered with other health services. We cannot account for health care utilization that occurs outside of SBHCs, such as school-based or third-party community-based mental health services. These limitations, however, are likely to bias our results toward the null hypothesis. Additional studies are needed to explore which delivery models are most effective for specific subgroups of students. Finally, we were not able to account for reasons for school absences, such as illness or suspensions, due to limitations of the data set.

Despite these limitations, our findings suggest that SBHC utilization plays a critical role in students’ educational engagement. These findings have major implications for future resource allocation within school districts, specifically for those whose funding is directly related to daily attendance counts. Investment in SBHCs and mental health services could be helpful for bringing in necessary funds to maintain and expand school services, increase resources to combat mental health stigma, refer students to appropriate supports, and practice trauma-informed teaching. While the newly passed Bipartisan Safer Communities Act does include measures to increase numbers of school-based mental health professionals and expand mental health services, there is also a need to invest in mental health training pipeline programs, given the critical shortage of school psychologists who are equipped to address the needs of students [28]. Ultimately, dedicated and continued investment in SBHCs may help to address health needs in children and adolescents and improve academic outcomes, particularly for underserved groups and students with specific mental health needs.

### Technical appendix

#### Proxy date assignment.

For those who did not visit an SBHC (i.e., the unexposed), we applied a proxy date for SBHC attendance [19]. The creation of this proxy date occurred as a multistage process. In stage 1, among those who visited an SBHC (i.e., the exposed) we ascertained the distribution of the combination of their sex and the month and year of the first appearance in the attendance dataset (i.e., their minimum date). Developing a matching scheme based on the month and year of first appearance in the attendance data was meant to ensure that potential secular trends in attendance in the exposed would be similar in the unexposed. In stage 2, we compared the observed relative frequencies for the combined sex/minimum date in the exposed group to those in the unexposed group. To ensure that the distribution in the unexposed would match those in the exposed, we multiplied the total samples size for the unexposed ($NU_{i}$) across all strata (i) by the relative frequency for this strata from the exposed ($NE_{i}$), see equation 1. This created a maximal sample size ($MMS_{i}$) that could be drawn from each stratum in the unexposed group that would preserve the relative frequencies of these matching variables in the exposed.


Eqn 1 $${\mathrm{Maximal}\;\mathrm{Sample}\;\mathrm{Size}}_{i}=\left(\sum_{i=1}^{n}\;NU_{i}\right)*\left(\frac{NE_{i}}{\sum_{i=1}^{n}\;NE_{i}}\right)$$

However, the maximal sample size calculated for each stratum could exceed the actual number of participants in the unexposed group that were available from those strata. As a result, in stage 3, we developed a scaling factor to allow us select the largest possible number of unexposed participants from each stratum while still preserving the distribution of the matching variables. The scaling factor (SF) was based on determining how many times larger the maximal sample size to pull from each stratum was compared to the available sample size in the unexposed, see equation 2. The maximal amount of this overestimation could then be used to rescale the maximal sample size to not exceed the available sample size within each strata, allowing us to generate the number of unexposed individuals to select ($NUS_{i}$) within each strata, see equation 3.


Eqn 2 $$\mathrm{Scaling}\;\mathrm{Factor}=max\left(\frac{MMS_{i}}{NU_{i}}\right)$$

Eqn 3 $$\mathrm{Number}\;\mathrm{of}\;\mathrm{Unexposed}\;\mathrm{to}\;{\mathrm{Select}}_{i}=\left(\frac{{\mathrm{MMS}}_{i}}{SF}\right)$$

With the number of unexposed individuals to select from each stratum determined, in stage 4, we randomly selected these individuals for inclusion into the analytic dataset. Using the observed distribution of the SBHC attendance dates (month and year) in the exposed group within each of the strata, we randomly assigned the proxy SBHC dates to the unexposed individuals. In this manner, the unexposed individuals were not only matched on the sex and minimum date in the attendance data, but also were assigned proxy dates that reflected the observed distribution of the SBHC visits. This ensured that sex differences and secular trends in attendance would be minimized in subsequent analyses. For assigning proxy dates in the mental health analyses, a single individual from the exposed group who visited an SBHC in June 2016 was removed from the analysis due to their influence on the scaling factor, which, if they were included, would have resulted in a reduction in the unexposed group sample size of 55,828.

#### Propensity score development.

In addition to matching the sex distribution and between those who did and did not visit an SBHC, we developed a propensity score weight based on race, sex, and their interaction in predicting and individuals use of an SBHC. This propensity score (ps) was converted to an inverse probability weight for each individual (j) based on the presence in the exposed group (1 = exposed; 0 = unexposed), see equation 4. All subsequent analyses utilized this weight.


Eqn 4 $$Inverse\;Probability\;Weight=\left(\frac{{\mathrm{exposed}}_{j}}{ps_{j}}\right)+\left(\frac{1\;-\;{\mathrm{exposed}}_{j}}{1\;-\;ps_{j}}\right)$$

## Supplementary Material

MMC1

MMC2

Supplementary Data

Supplementary data related to this article can be found at https://doi.org/10.1016/j.jadohealth.2023.07.012.

## Figures and Tables

**Table 1:** Characteristics of SBHC users and non-users

	First SBHC visit		First SBHC mental health visit	
	Nonusers N = 230,046	Users N = 14,030	Nonusers N = 111,448	Users N = 983

Sex, % (n)				
Female	56.6 (130,173)	56.5 (7,929)	54.7 (60,937)	54.5 (537)
Male	43.4 (99,873)	43.5 (6,101)	45.3 (50,511)	45.5 (446)
Race/Ethnicity, % (n)				
Asian	5.9 (14,087)	5.9 (358)	5.9 (6,636)	5.6 (19)
Black	6.0 (12,815)	6.2 (1,848)	5.4 (5,975)	5.7 (117)
Latine	76.6 (175,522)	76.4 (11,360)	77.0 (85,732)	77.8 (815)
White	10.5 (25,398)	10.5 (323)	10.9 (12,269)	10.4 (25)
Two or more	0.2 (573)	0.3 (21)	0.0 (0)	0.0 (0)
Unknown	0.7 (1,651)	0.7 (120)	0.8 (836)	0.5 (7)

Weighted percentages are reported. We developed a maximal sample size that could be drawn from each stratum in the unexposed group that would preserve the relative frequencies of the matching variables in the exposed.

**Table 2:** Difference in attendance trajectory following an SBHC visit and SBHC mental health visit

	Pre slope (95% CI)	Post slope (95% CI)	Difference (95% CI)	Difference-in-difference	*p* value for diff-in-diff

SBHC Users	−0.71 (−0.85, −0.58)	−0.05 (−0.25, 0.14)	0.66 (0.42, 0.91)	0.65	<.001
Control (non-users)	−0.18 (−0.20, −0.15)	−0.17 (−0.20, −0.14)	0.01 (−0.03, 0.04)		
SBHC Mental Health Users	−1.02 (−1.58, −0.46)	1.44 (0.80, 2.09)	2.46 (1.52, 3.40)	2.33	<.001
Control (non-users)	−0.25 (−0.28, −0.22)	−0.11 (−0.16, −0.07)	0.13 (0.08, 0.19)		

Slope may be interpreted as the rate of change in the percent of full-days present over nine months (the approximate length of the school year).

## References

[1] Love HE, Schlitt J, Soleimanpour S, Twenty years of school-based health care growth and expansion. Health Aff 2019;38:755–64. 10.1377/hlthaff.2018.05472 31059359

[2] Knopf JA, Finnie RK, Peng Y, School-based health centers to advance health equity: A community guide systematic review. Am J Prev Med 2016;51:114–26. 27320215 10.1016/j.amepre.2016.01.009 PMC5759331

[3] Allison MA, Attisha E. The link between school attendance and good health. Pediatrics 2019;143:e20183648. 30835245 10.1542/peds.2018-3648

[4] Arenson M, Hudson PJ, Lee N, Lai B. The evidence on school-based health centers: A review. Global pediatric health 2019;6:1–10. 10.1177/2333794X19828745 PMC6381423 30815514

[5] Racine N, McArthur BA, Cooke JE, Global prevalence of depressive and anxiety symptoms in children and adolescents during COVID-19: A meta-analysis. JAMA Pediatr 2021;175:1142–50. 34369987 10.1001/jamapediatrics.2021.2482 PMC8353576

[6] Dalsgaard S, McGrath J, Østergaard SD, Association of mental disorder in childhood and adolescence with subsequent educational achievement. JAMA Psychiatr 2020;77:797–805. 10.1001/jamapsychiatry.2020.0217 PMC7097843 32211833

[7] Bains RM, Diallo AF. Mental health services in school-based health centers: Systematic review. J Sch Nurs 2016;32:8–19. 26141707 10.1177/1059840515590607

[8] Ali MM, West K, Teich JL, Utilization of mental health services in educational setting by adolescents in the United States. J Sch Health 2019;89:393–401. 30883761 10.1111/josh.12753

[9] Lawrence D, Dawson V, Houghton S, Impact of mental disorders on attendance at school. Aust J Educ 2019;63:5–21.

[10] Eklund K, Burns MK, Oyen K, Addressing chronic absenteeism in schools: A meta-analysis of evidence-based interventions. Sch Psychol Rev 2022;51:95–111.

[11] Bersamin M, Garbers S, Gold MA, Measuring success: Evaluation designs and approaches to assessing the impact of school-based health centers. J Adolesc Health 2016;58:3–10. 26707224 10.1016/j.jadohealth.2015.09.018 PMC4693147

[12] Van Cura M The relationship between school-based health centers, rates of early dismissal from school, and loss of seat time. J Sch Health 2010;80:371–7. 20618619 10.1111/j.1746-1561.2010.00516.x

[13] Webber MP, Carpiniello KE, Oruwariye T, Burden of asthma in inner-city elementary schoolchildren: Do school-based health centers make a difference? Arch Pediatr Adolesc Med 2003;157:125–9. 12580680 10.1001/archpedi.157.2.125

[14] Walker SC, Kerns SE, Lyon AR, Impact of school-based health center use on academic outcomes. J Adolesc Health 2010;46:251–7. 20159502 10.1016/j.jadohealth.2009.07.002

[15] Lê-Scherban F, Roux AVD, Li Y, Morgenstern H. Does academic achievement during childhood and adolescence benefit later health? Ann Epidemiol 2014;24:344–55. 24792585 10.1016/j.annepidem.2014.02.008 PMC4037140

[16] Thomas CL, Price OA, Phillippi S, Wennerstrom A. School-based health centers, academic achievement, and school discipline: A systematic review of the literature. Child Youth Serv Rev 2020;118:105467.

[17] Gallardo M, Zepeda A, Biely C, School-based health center utilization during COVID-19 pandemic-related school closures. J Sch Health 2022;92:1045–50. 35945893 10.1111/josh.13226 PMC9538881

[18] Schroeder AR, Dahlen A, Purington N, Healthcare utilization in children across the care continuum during the COVID-19 pandemic. PLoS One 2022;17:e0276461. 36301947 10.1371/journal.pone.0276461 PMC9612476

[19] Harvey RA, Jankus DAD, Mosley DG. Random assignment of proxy event dates to unexposed individuals in observational studies: an automated technique using SAS^®^. Midwest SAS Users Group (MSUG); 2012.

[20] Juszczak L, Melinkovich P, Kaplan D. Use of health and mental health services by adolescents across multiple delivery sites. J Adolesc Health 2003;32:108–18. 12782449 10.1016/s1054-139x(03)00073-9

[21] Hsu J, Qin X, Beavers SF, Mirabelli MC. Asthma-related school absenteeism, morbidity, and modifiable factors. Am J Prev Med 2016;51:23–32. 26873793 10.1016/j.amepre.2015.12.012 PMC4914465

[22] School connectedness: Strategies for increasing protective factors among youth. In: Centers for Disease Control and Prevention. US Department of Health and Human Services; 2009.

[23] Gruber JA, Anderson-Carpenter KD, McNall M, Clark SL. Understanding the longitudinal impact of school-based health centers on student attendance. Child Youth Care Forum 2022;52:331–50.

[24] Statement of Administration policy: Bipartisan safer communities act. Statement of Administration policy: Bipartisan safer communities act. Executive office of the president. Available at: https://www.whitehouse.gov/wp-content/uploads/2022/06/Bipartisan-Safer-Communities-Act-SAP-1.pdf. Accessed February 7, 2023.

[25] Ferenchak KS, Trieu SL, Franco R, Beyond Co-Location: Development of a school health integration measure. J Sch Health 2021;91:970–80. 34636051 10.1111/josh.13088

[26] Moore A, Stapley E, Hayes D, Barriers and facilitators to sustaining school-based mental health and wellbeing interventions: A systematic review. Int J Environ Res Publ Health 2022;19:3587. 10.3390/ijerph19063587 PMC8949982 35329276

[27] What are community schools? National education association. Available at: https://www.nea.org/student-success/great-public-schools/community-schools. Accessed March 5, 2023

[28] Castillo JM, Curtis MJ, Tan SY. Personnel needs in school psychology: A 10-year follow-up study on predicted personnel shortages. Psychol Sch 2014;51:832–49.

