# Supplementary material for: School Attendance Following Receipt of Care From a School-Based Health Center
Source: J Adolesc Health. Author manuscript; Available in PMC 2026 Jun 10. (PMC13251883; doi:10.1016/j.jadohealth.2023.07.012)
Supplement: MMC1 [file NIHMS2174531-supplement-MMC1.docx]

**Appendix Table A1. Sensitivity Analysis Including All Attendance Observations**

|  | Pre Slope (95% CI) | Post Slope (95% CI) | Difference  (95% CI) | Difference-in-difference | P-value for diff-in-diff |
| --- | --- | --- | --- | --- | --- |
| SBHC Users | -0.51  (-0.62, -0.41) | 0.20  (0.02, 0.38) | 0.71  (0.50, 0.93) | 0.60 | <.001 |
| Control (non-users) | -0.20  (-0.22, -0.18) | -0.08  (-0.10, -0.06) | 0.12  (0.09, 0.15) |  |  |
| SBHC Mental Health Users | -0.67  (-0.98, -0.36) | 1.44  (0.90, 1.98) | 2.11  (1.43, 2.78) | 1.89 | <.001 |
| Control (non-users) | -0.24  (-0.26, -0.22) | -0.02  (-0.06, 0.02) | 0.22  (0.17, 0.27) |  |  |
