# Supplementary material for: School Attendance Following Receipt of Care From a School-Based Health Center
Source: J Adolesc Health. Author manuscript; Available in PMC 2026 Jun 10. (PMC13251883; doi:10.1016/j.jadohealth.2023.07.012)
Supplement: MMC2 [file NIHMS2174531-supplement-MMC2.docx]

**Appendix Table A2. Sensitivity Analysis Including Those with 6 Months of Data within 12 Months before and after Attending the SBHC**

|  | Pre Slope (95% CI) | Post Slope (95% CI) | Difference  (95% CI) | Difference-in-difference | P-value for diff-in-diff |
| --- | --- | --- | --- | --- | --- |
| SBHC Users | -1.08  (-1.20, -0.97) | -0.40  (-0.67, -0.13) | 0.68  (0.40, 0.96) | 0.44 | .003 |
| Control (non-users) | -0.45  (-0.47, -0.43) | -0.21  (-0.24, -0.17) | 0.25  (0.21, 0.29) |  |  |
| SBHC Mental Health Users | -1.54  (-2.00, -1.09) | 0.79  (-0.21, 1.79) | 2.33  (1.18, 3.49) | 1.94 | .001 |
| Control (non-users) | -0.54  (-0.57, -0.51) | -0.15  (-0.20, -0.10) | 0.39  (0.33, 0.45) |  |  |
